# Future themes of mathematics education research: an international survey before and during the pandemic

**DOI:** 10.1007/s10649-021-10049-w

**Published:** 2021-04-06

**Authors:** Arthur Bakker, Jinfa Cai, Linda Zenger

**Affiliations:** 1 Utrecht University, Utrecht, Netherlands; 2 University of Delaware, Newark, DE USA

**Keywords:** COVID-19, Grand challenges, Pandemic, Mathematics education research, Research agenda

## Abstract

Before the pandemic (2019), we asked: *On what themes should research in mathematics education focus in the coming decade?* The 229 responses from 44 countries led to eight themes plus considerations about mathematics education research itself. The themes can be summarized as teaching approaches, goals, relations to practices outside mathematics education, teacher professional development, technology, affect, equity, and assessment. During the pandemic (November 2020), we asked respondents: *Has the pandemic changed your view on the themes of mathematics education research for the coming decade? If so, how?* Many of the 108 respondents saw the importance of their original themes reinforced (45), specified their initial responses (43), and/or added themes (35) (these categories were not mutually exclusive). Overall, they seemed to agree that the pandemic functions as a magnifying glass on issues that were already known, and several respondents pointed to the need to think ahead on how to organize education when it does not need to be online anymore. We end with a list of research challenges that are informed by the themes and respondents’ reflections on mathematics education research.

## An international survey in two rounds

Around the time when *Educational Studies in Mathematics* (ESM) and the *Journal for Research in Mathematics Education* (JRME) were celebrating their 50th anniversaries, Arthur Bakker (editor of ESM) and Jinfa Cai (editor of JRME) saw a need to raise the following future-oriented question for the field of mathematics education research:

*Q2019: On what themes should research in mathematics education focus in the coming decade?*

To that end, we administered a survey with just this one question between June 17 and October 16, 2019.

When we were almost ready with the analysis, the COVID-19 pandemic broke out, and we were not able to present the results at the conferences we had planned to attend (NCTM and ICME in 2020). Moreover, with the world shaken up by the crisis, we wondered if colleagues in our field might think differently about the themes formulated for the future due to the pandemic. Hence, on November 26, 2020, we asked a follow-up question to those respondents who in 2019 had given us permission to approach them for elaboration by email:

*Q2020: Has the pandemic changed your view on the themes of mathematics education research for the coming decade? If so, how?*

In this paper, we summarize the responses to these two questions. Similar to Sfard’s (2005) approach, we start by synthesizing the voices of the respondents before formulating our own views. Some colleagues put forward the idea of formulating a list of key themes or questions, similar to the 23 unsolved mathematical problems that David Hilbert published around 1900 (cf. Schoenfeld, 1999). However, mathematics and mathematics education are very different disciplines, and very few people share Hilbert’s formalist view on mathematics; hence, we do not want to suggest that we could capture the key themes of mathematics education in a similar way. Rather, our overview of themes drawn from the survey responses is intended to summarize what is valued in our global community at the time of the surveys. Reasoning from these themes, we end with a list of research challenges that we see worth addressing in the future (cf. Stephan et al., 2015).

## Methodological approach

### Themes for the coming decade (2019)

We administered the 1-question survey through email lists that we were aware of (e.g., Becker, ICME, PME) and asked mathematics education researchers to spread it in their national networks. By October 16, 2019, we had received 229 responses from 44 countries across 6 continents (Table 1). Although we were happy with the larger response than Sfard (2005) received (74, with 28 from Europe), we do not know how well we have reached particular regions, and if potential respondents might have faced language or other barriers. We did offer a few Chinese respondents the option to write in Chinese because the second author offered to translate their emails into English. We also received responses in Spanish, which were translated for us.

**Table 1:** Numbers of responses per continent (2019)

Continent (# of countries)	Countries (# of responses)	# of responses
Asia (12)	China (39), Israel (14), India (9), Japan (4), Indonesia (3), Russia (3), Iran (2), Taiwan (2), United Arab Emirates (2), Bhutan (1), Philippines (1), Turkey (1)	81
Europe (15)	UK (17.5), Germany (10), the Netherlands (10), Spain (9), Italy (7), Austria (3), Sweden (3), France (2), Hungary (2), Ireland (2), Czech Republic (1), Denmark (1), Iceland (1), Norway (1), Slovenia (1)	70.5
North America (3)	USA (22.5); Canada (6); Mexico (1)	29.5
Africa (10)	Kenya (8), South Africa (8), Namibia (4), Algeria (1), Egypt (1), Eswatini (1), Ghana (1), Morocco (1), Nigeria (1), Uganda (1)	27
Oceania (2)	Australia (7); New Zealand (4)	11
South America (2)	Brazil (5); Chile (5)	10
Totals: 6	44	229

*Note*: When a respondent filled in two countries on two continents, we attributed half to one and the other half to the other continent

Ethical approval was given by the Ethical Review Board of the Faculties of Science and Geo-science of Utrecht University (Bèta L-19247). We asked respondents to indicate if they were willing to be quoted by name and if we were allowed to approach them for subsequent information. If they preferred to be named, we mention their name and country; otherwise, we write “anonymous.” In our selection of quotes, we have focused on content, not on where the response came from. On March 2, 2021, we approached all respondents who were quoted to double-check if they agreed to be quoted and named. One colleague preferred the quote and name to be deleted; three suggested small changes in wording; the others approved.

On September 20, 2019, the three authors met physically at Utrecht University to analyze the responses. After each individual proposal, we settled on a joint list of seven main themes (the first seven in Table 2), which were neither mutually exclusive nor exhaustive. The third author (Zenger, then still a student in educational science) next color coded all parts of responses belonging to a category. These formed the basis for the frequencies and percentages presented in the tables and text. The first author (Bakker) then read all responses categorized by a particular code to identify and synthesize the main topics addressed within each code. The second author (Cai) read all of the survey responses and the response categories, and commented. After the initial round of analysis, we realized it was useful to add an eighth theme: assessment (including evaluation).

**Table 2:** Percentages of responses mentioned in each theme (2019)

	Theme	%
1	Approaches to teaching	64
2	Goals of mathematics education	54
3	Relation of mathematics education with other practices	36
4	Professional development of teachers	23
5	Technology	22
6	Equity, diversity, inclusion	20
7	Affect	17
8	Assessment	9

*Note.* Percentages do not add up to 100, because many respondents mentioned multiple themes

Moreover, given that a large number of respondents made comments about mathematics education research itself, we decided to summarize these separately. For analyzing this category of research, we used the following four labels to distinguish types of comments on our discipline of mathematics education research: theory, methodology, self-reflection (including ethical considerations), interdisciplinarity, and transdisciplinarity. We then summarized the responses per type of comment.

**Fig. 1:**
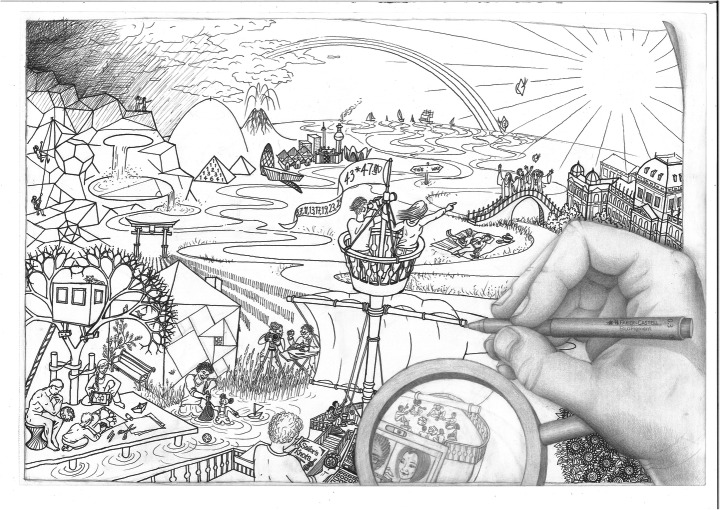
Artistic impression of the future themes

It has been a daunting and humbling experience to study the huge coverage and diversity of topics that our colleagues care about. Any categorization felt like a reduction of the wealth of ideas, and we are aware of the risks of “sorting things out” (Bowker & Star, 2000), which come with foregrounding particular challenges rather than others (Stephan et al., 2015). Yet the best way to summarize the bigger picture seemed by means of clustering themes and pointing to their relationships. As we identified these eight themes of mathematics education research for the future, a recurring question during the analysis was how to represent them. A list such as Table 2 does not do justice to the interrelations between the themes. Some relationships are very clear, for example, educational approaches (theme 2) working toward educational or societal goals (theme 1). Some themes are pervasive; for example, equity and (positive) affect are both things that educators want to achieve but also phenomena that are at stake during every single moment of learning and teaching. Diagrams we considered to represent such interrelationships were either too specific (limiting the many relevant options, e.g., a star with eight vertices that only link pairs of themes) or not specific enough (e.g., a Venn diagram with eight leaves such as the iPhone symbol for photos). In the end, we decided to use an image and collaborated with Elisabeth Angerer (student assistant in an educational sciences program), who eventually made the drawing in Fig. 1 to capture themes in their relationships.

### Has the pandemic changed your view? (2020)

On November 26, 2020, we sent an email to the colleagues who responded to the initial question and who gave permission to be approached by email. We cited their initial response and asked: “Has the pandemic changed your view on the themes of mathematics education research for the coming decade? If so, how?” We received 108 responses by January 12, 2021. The countries from which the responses came included China, Italy, and other places that were hit early by the COVID-19 virus. The length of responses varied from a single word response (“no”) to elaborate texts of up to 2215 words. Some people attached relevant publications. The median length of the responses was 87 words, with a mean length of 148 words and SD = 242. Zenger and Bakker classified them as “no changes” (9 responses) or “clearly different views” (8); the rest of the responses saw the importance of their initial themes reinforced (45), specified their initial responses (43), or added new questions or themes (35). These last categories were not mutually exclusive, because respondents could first state that they thought the initial themes were even more relevant than before and provide additional, more specified themes. We then used the same themes that had been identified in the first round and identified what was stressed or added in the 2020 responses.

## The themes

The most frequently mentioned theme was what we labeled approaches to teaching (64% of the respondents, see Table 2). Next was the theme of goals of mathematics education on which research should shed more light in the coming decade (54%). These goals ranged from specific educational goals to very broad societal ones. Many colleagues referred to mathematics education’s relationships with other practices (communities, institutions…) such as home, continuing education, and work. Teacher professional development is a key area for research in which the other themes return (what should students learn, how, how to assess that, how to use technology and ensure that students are interested?). Technology constitutes its own theme but also plays a key role in many other themes, just like affect. Another theme permeating other ones is what can be summarized as equity, diversity, and inclusion (also social justice, anti-racism, democratic values, and several other values were mentioned). These values are not just societal and educational goals but also drivers for redesigning teaching approaches, using technology, working on more just assessment, and helping learners gain access, become confident, develop interest, or even love for mathematics. To evaluate if approaches are successful and if goals have been achieved, assessment (including evaluation) is also mentioned as a key topic of research.

In the 2020 responses, many wise and general remarks were made. The general gist is that the pandemic (like earlier crises such as the economic crisis around 2008–2010) functioned as a magnifying glass on themes that were already considered important. Due to the pandemic, however, systemic societal and educational problems were said to have become better visible to a wider community, and urge us to think about the potential of a “new normal.”

### Approaches to teaching

We distinguish specific teaching strategies from broader curricular topics.

#### Teaching strategies

There is a widely recognized need to further design and evaluate various teaching approaches. Among the teaching strategies and types of learning to be promoted that were mentioned in the survey responses are collaborative learning, critical mathematics education, dialogic teaching, modeling, personalized learning, problem-based learning, cross-curricular themes addressing the bigger themes in the world, embodied design, visualization, and interleaved learning. Note, however, that students can also enhance their mathematical knowledge independently from teachers or parents through web tutorials and YouTube videos.

Many respondents emphasized that teaching approaches should do more than promote cognitive development. How can teaching be entertaining or engaging? How can it contribute to the broader educational goals of developing students’ identity, contribute to their empowerment, and help them see the value of mathematics in their everyday life and work? We return to affect in Section 3.7.

In the 2020 responses, we saw more emphasis on approaches that address modeling, critical thinking, and mathematical or statistical literacy. Moreover, respondents stressed the importance of promoting interaction, collaboration, and higher order thinking, which are generally considered to be more challenging in distance education. One approach worth highlighting is challenge-based education (cf. Johnson et al. 2009), because it takes big societal challenges as mentioned in the previous section as its motivation and orientation.

#### Curriculum

Approaches by which mathematics education can contribute to the aforementioned goals can be distinguished at various levels. Several respondents mentioned challenges around developing a coherent mathematics curriculum, smoothing transitions to higher school levels, and balancing topics, and also the typical overload of topics, the influence of assessment on what is taught, and what teachers can teach. For example, it was mentioned that mathematics teachers are often not prepared to teach statistics. There seems to be little research that helps curriculum authors tackle some of these hard questions as well as how to monitor reform (cf. Shimizu & Vithal, 2019). Textbook analysis is mentioned as a necessary research endeavor. But even if curricula within one educational system are reasonably coherent, how can continuity between educational systems be ensured (cf. Jansen et al., 2012)?

In the 2020 responses, some respondents called for free high-quality curriculum resources. In several countries where Internet access is a problem in rural areas, a shift can be observed from online resources to other types of media such as radio and TV.

### Goals of mathematics education

The theme of approaches is closely linked to that of the theme of goals. For example, as Fulvia Furinghetti (Italy) wrote: “It is widely recognized that critical thinking is a fundamental goal in math teaching. Nevertheless it is still not clear how it is pursued in practice.” We distinguish broad societal and more specific educational goals. These are often related, as Jane Watson (Australia) wrote: “If Education is to solve the social, cultural, economic, and environmental problems of today’s data-driven world, attention must be given to preparing students to interpret the data that are presented to them in these fields.”

#### Societal goals

Respondents alluded to the need for students to learn to function in the economy and in society more broadly. Apart from instrumental goals of mathematics education, some emphasized goals related to developing as a human being, for instance learning to see the mathematics in the world and develop a relation with the world. Mathematics education in these views should empower students to combat anti-expertise and post-fact tendencies. Several respondents mentioned even larger societal goals such as avoiding extinction as a human species and toxic nationalism, resolving climate change, and building a sustainable future.

In the second round of responses (2020), we saw much more emphasis on these bigger societal issues. The urgency to orient mathematics education (and its research) toward resolving these seemed to be felt more than before. In short, it was stressed that our planet needs to be saved. The big question is what role mathematics education can play in meeting these challenges.

#### Educational goals

Several respondents expressed a concern that the current goals of mathematics education do not reflect humanity’s and societies’ needs and interests well. Educational goals to be stressed more were mathematical literacy, numeracy, critical, and creative thinking—often with reference to the changing world and the planet being at risk. In particular, the impact of technology was frequently stressed, as this may have an impact on what people need to learn (cf. Gravemeijer et al., 2017). If computers can do particular things much better than people, what is it that students need to learn?

Among the most frequently mentioned educational goals for mathematics education were statistical literacy, computational and algorithmic thinking, artificial intelligence, modeling, and data science. More generally, respondents expressed that mathematics education should help learners deploy evidence, reasoning, argumentation, and proof. For example, Michelle Stephan (USA) asked:

What mathematics content should be taught today to prepare students for jobs of the future, especially given growth of the digital world and its impact on a global economy? All of the mathematics content in K-12 can be accomplished by computers, so what mathematical procedures become less important and what domains need to be explored more fully (e.g., statistics and big data, spatial geometry, functional reasoning, etc.)?

One challenge for research is that there is no clear methodology to arrive at relevant and feasible learning goals. Yet there is a need to choose and formulate such goals on the basis of research (cf. Van den Heuvel-Panhuizen, 2005).

Several of the 2020 responses mentioned the sometimes problematic way in which numbers, data, and graphs are used in the public sphere (e.g., Ernest, 2020; Kwon et al., 2021; Yoon et al., 2021). Many respondents saw their emphasis on relevant educational goals reinforced, for example, statistical and data literacy, modeling, critical thinking, and public communication. A few pandemic-specific topics were mentioned, such as exponential growth.

### Relation of mathematics education to other practices

Many responses can be characterized as highlighting boundary crossing (Akkerman & Bakker, 2011) with disciplines or communities outside mathematics education, such as in science, technology, engineering, art, and mathematics education (STEM or STEAM); parents or families; the workplace; and leisure (e.g., drama, music, sports). An interesting example was the educational potential of mathematical memes—“humorous digital objects created by web users copying an existing image and overlaying a personal caption” (Bini et al., 2020, p. 2). These boundary crossing-related responses thus emphasize the movements and connections between mathematics education and other practices.

In the 2020 responses, we saw that during the pandemic, the relationship between school and home has become much more important, because most students were (and perhaps still are) learning at home. Earlier research on parental involvement and homework (Civil & Bernier, 2006; de Abreu et al., 2006; Jackson, 2011) proves relevant in the current situation where many countries are still or again in lockdown. Respondents pointed to the need to monitor students and their work and to promote self-regulation. They also put more stress on the political, economic, and financial contexts in which mathematics education functions (or malfunctions, in many respondents’ views).

### Teacher professional development

Respondents explicitly mentioned teacher professional development as an important domain of mathematics education research (including teacher educators’ development). For example, Loide Kapenda (Namibia) wrote, “I am supporting UNESCO whose idea is to focus on how we prepare teachers for the future we want.” (e.g., UNESCO, 2015) And, Francisco Rojas (Chile) wrote:

Although the field of mathematics education is broad and each time faced with new challenges (socio-political demands, new intercultural contexts, digital environments, etc.), all of them will be handled at school by the mathematics teacher, both in primary as well as in secondary education. Therefore, from my point of view, pre-service teacher education is one of the most relevant fields of research for the next decade, especially in developing countries.

It is evident from the responses that teaching mathematics is done by a large variety of people, not only by people who are trained as primary school teachers, secondary school mathematics teachers, or mathematicians but also parents, out-of-field teachers, and scientists whose primary discipline is not mathematics but who do use mathematics or statistics. How teachers of mathematics are trained varies accordingly. Respondents frequently pointed to the importance of subject-matter knowledge and particularly noted that many teachers seem ill-prepared to teach statistics (e.g., Lonneke Boels, the Netherlands).

Key questions were raised by several colleagues: “How to train mathematics teachers with a solid foundation in mathematics, positive attitudes towards mathematics teaching and learning, and wide knowledge base linking to STEM?” (anonymous); “What professional development, particularly at the post-secondary level, motivates changes in teaching practices in order to provide students the opportunities to engage with mathematics and be successful?” (Laura Watkins, USA); “How can mathematics educators equip students for sustainable, equitable citizenship? And how can mathematics education equip teachers to support students in this?” (David Wagner, Canada)

In the 2020 responses, it was clear that teachers are incredibly important, especially in the pandemic era. The sudden change to online teaching means that

higher requirements are put forward for teachers’ educational and teaching ability, especially the ability to carry out education and teaching by using information technology should be strengthened. Secondly, teachers’ ability to communicate and cooperate has been injected with new connotation. (Guangming Wang, China)

It is broadly assumed that education will stay partly online, though more so in higher levels of education than in primary education. This has implications for teachers, for instance, they will have to think through how they intend to coordinate teaching on location and online. Hence, one important focus for professional development is the use of technology.

### Technology

Technology deserves to be called a theme in itself, but we want to emphasize that it ran through most of the other themes. First of all, some respondents argued that, due to technological advances in society, the societal and educational *goals* of mathematics education need to be changed (e.g., computational thinking to ensure employability in a technological society). Second, responses indicated that the changed goals have implications for the *approaches* in mathematics education. Consider the required curriculum reform and the digital tools to be used in it. Students do not only need to learn to use technology; the technology can also be used to learn mathematics (e.g., visualization, embodied design, statistical thinking). New technologies such as 3D printing, photo math, and augmented and virtual reality offer new opportunities for learning. Society has changed very fast in this respect. Third, technology is suggested to assist in establishing *connections with other practices*, such as between school and home, or vocational education and work, even though there is a great disparity in how successful these connections are.

In the 2020 responses, there was great concern about the current digital divide (cf. Hodgen et al., 2020). The COVID-19 pandemic has thus given cause for mathematics education research to understand better how connections across educational and other practices can be improved with the help of technology. Given the unequal distribution of help by parents or guardians, it becomes all the more important to think through how teachers can use videos and quizzes, how they can monitor their students, how they can assess them (while respecting privacy), and how one can compensate for the lack of social, gestural, and embodied interaction that is possible when being together physically.

Where mobile technology was considered very innovative before 2010, smartphones have become central devices in mathematics education in the pandemic with its reliance on distance learning. Our direct experience showed that phone applications such as WhatsApp and WeChat have become key tools in teaching and learning mathematics in many rural areas in various continents where few people have computers (for a report on podcasts distributed through WhatsApp, community loudspeakers, and local radio stations in Colombia, see Saenz et al., 2020).

### Equity, diversity, and inclusion

Another cross-cutting theme can be labeled “equity, diversity, and inclusion.” We use this triplet to cover any topic that highlights these and related human values such as equality, social and racial justice, social emancipation, and democracy that were also mentioned by respondents (cf. Dobie & Sherin, 2021). In terms of educational *goals*, many respondents stressed that mathematics education should be for all students, including those who have special needs, who live in poverty, who are learning the instruction language, who have a migration background, who consider themselves LGBTQ+, have a traumatic or violent history, or are in whatever way marginalized. There is broad consensus that everyone should have access to high-quality mathematics education. However, as Niral Shah (USA) notes, less attention has been paid to “how phenomena related to social markers (e.g., race, class, gender) interact with phenomena related to the teaching and learning of mathematical content.”

In terms of *teaching approaches*, mathematics education is characterized by some respondents from particular countries as predominantly a white space where some groups feel or are excluded (cf. Battey, 2013). There is a general concern that current practices of teaching mathematics may perpetuate inequality, in particular in the current pandemic. In terms of *assessment*, mathematics is too often used or experienced as a gatekeeper rather than as a powerful resource (cf. Martin et al., 2010). Steve Lerman (UK) “indicates that understanding how educational opportunities are distributed inequitably, and in particular how that manifests in each end every classroom, is a prerequisite to making changes that can make some impact on redistribution.” A key research aim therefore is to understand what excludes students from learning mathematics and what would make mathematics education more inclusive (cf. Roos, 2019). And, what does *professional development* of teachers that promotes equity look like?

In 2020, many respondents saw their emphasis on equity and related values reinforced in the current pandemic with its risks of a digital divide, unequal access to high-quality mathematics education, and unfair distribution of resources. A related future research theme is how the so-called widening achievement gaps can be remedied (cf. Bawa, 2020). However, warnings were also formulated that thinking in such deficit terms can perpetuate inequality (cf. Svensson et al., 2014). A question raised by Dor Abrahamson (USA) is, “What roles could digital technology play, and in what forms, in restoring justice and celebrating diversity?”

### Affect

Though entangled with many other themes, affect is also worth highlighting as a theme in itself. We use the term affect in a very broad sense to point to psychological-social phenomena such as emotion, love, belief, attitudes, interest, curiosity, fun, engagement, joy, involvement, motivation, self-esteem, identity, anxiety, alienation, and feeling of safety (cf. Cobb et al., 2009; Darragh, 2016; Hannula, 2019; Schukajlow et al., 2017). Many respondents emphasized the importance of studying these constructs in relation to (and not separate from) what is characterized as cognition. Some respondents pointed out that affect is not just an individual but also a social phenomenon, just like learning (cf. Chronaki, 2019; de Freitas et al., 2019; Schindler & Bakker, 2020).

Among the educational *goals* of mathematics education, several participants mentioned the need to generate and foster interest in mathematics. In terms of *approaches*, much emphasis was put on the need to avoid anxiety and alienation and to engage students in mathematical activity.

In the 2020 responses, more emphasis was put on the concern about alienation, which seems to be of special concern when students are socially distanced from peers and teachers as to when teaching takes place only through *technology*. What was reiterated in the 2020 responses was the importance of students’ sense of belonging in a mathematics classroom (cf. Horn, 2017)—a topic closely related to the theme of equity, diversity, and inclusion discussed before.

### Assessment

Assessment and evaluation were not often mentioned explicitly, but they do not seem less important than the other related themes. A key challenge is to assess what we value rather than valuing what we assess. In previous research, the assessment of individual students has received much attention, but what seems to be neglected is the evaluation of curricula. As Chongyang Wang (China) wrote, “How to evaluate the curriculum reforms. When we pay much energy in reforming our education and curriculum, do we imagine how to ensure it will work and there will be pieces of evidence found after the new curricula are carried out? How to prove the reforms work and matter?” (cf. Shimizu & Vithal, 2019)

In the 2020 responses, there was an emphasis on assessment at a distance. Distance education generally is faced with the challenge of evaluating student work, both formatively and summatively. We predict that so-called e-assessment, along with its privacy challenges, will generate much research interest in the near future (cf. Bickerton & Sangwin, 2020).

## Mathematics education research itself

Although we only asked for future themes, many respondents made interesting comments about research in mathematics education and its connections with other disciplines and practices (such as educational practice, policy, home settings). We have grouped these considerations under the subheadings of theory, methodology, reflection on our discipline, and interdisciplinarity and transdisciplinarity. As with the previous categorization into themes, we stress that these four types are not mutually exclusive as theoretical and methodological considerations can be intricately intertwined (Radford, 2008).

### Theory

Several respondents expressed their concern about the fragmentation and diversity of theories used in mathematics education research (cf. Bikner-Ahsbahs & Prediger, 2014). The question was raised how mathematics educators can “work together to obtain valid, reliable, replicable, and useful findings in our field” and “How, as a discipline, can we encourage sustained research on core questions using commensurable perspectives and methods?” (Keith Weber, USA). One wish was “comparing theoretical perspectives for explanatory power” (K. Subramaniam, India). At the same time, it was stressed that “we cannot continue to pretend that there is just one culture in the field of mathematics education, that all the theoretical framework may be applied in whichever culture and that results are universal” (Mariolina Bartolini Bussi, Italy). In addition, the wish was expressed to deepen theoretical notions such as numeracy, equity, and justice as they play out in mathematics education.

### Methodology

Many methodological approaches were mentioned as potentially useful in mathematics education research: randomized studies, experimental studies, replication, case studies, and so forth. Particular attention was paid to “complementary methodologies that bridge the ‘gap’ between mathematics education research and research on mathematical cognition” (Christian Bokhove, UK), as, for example, done in Gilmore et al. (2018). Also, approaches were mentioned that intend to bridge the so-called gap between educational practice and research, such as lesson study and design research. For example, Kay Owens (Australia) pointed to the challenge of studying cultural context and identity: “Such research requires a multi-faceted research methodology that may need to be further teased out from our current qualitative (e.g., ethnographic) and quantitative approaches (‘paper and pencil’ (including computing) testing). Design research may provide further possibilities.”

Francisco Rojas (Chile) highlighted the need for more longitudinal and cross-sectional research, in particular in the context of teacher professional development:

It is not enough to investigate what happens in pre-service teacher education but understand what effects this training has in the first years of the professional career of the new teachers of mathematics, both in primary and secondary education. Therefore, increasingly more longitudinal and cross-sectional studies will be required to understand the complexity of the practice of mathematics teachers, how the professional knowledge that articulates the practice evolves, and what effects have the practice of teachers on the students’ learning of mathematics.

### Reflection on our discipline

Calls were made for critical reflection on our discipline. One anonymous appeal was for more self-criticism and scientific modesty: Is research delivering, or is it drawing away good teachers from teaching? Do we do research primarily to help improve mathematics education or to better understand phenomena? (cf. Proulx & Maheux, 2019) The general gist of the responses was a sincere wish to be of value to the world and mathematics education more specifically and not only do “research for the sake of research” (Zahra Gooya, Iran). David Bowers (USA) expressed several reflection-inviting views about the nature of our discipline, for example:

- We must normalize (and expect) the full taking up the philosophical and theoretical underpinnings of all of our work (even work that is not considered “philosophical”). Not doing so leads to uncritical analysis and implications.
- We must develop norms wherein it is considered embarrassing to do “uncritical” research.
- There is no such thing as “neutral.” Amongst other things, this means that we should be cultivating norms that recognize the inherent political nature of all work, and norms that acknowledge how superficially “neutral” work tends to empower the oppressor.
- We must recognize the existence of but not cater to the fragility of privilege.

In terms of *what* is studied, some respondents felt that the mathematics education research “literature has been moving away from the original goals of mathematics education. We seem to have been investigating everything but the actual learning of important mathematics topics.” (Lyn English, Australia) In terms of the *nature* of our discipline, Taro Fujita (UK) argued that our discipline can be characterized as a design science, with designing mathematical learning environments as the core of research activities (cf. Wittmann, 1995).

A tension that we observe in different views is the following: On the one hand, mathematics education research has its origin in helping teachers teach particular content better. The need for such so-called didactical, topic-specific research is not less important today but perhaps less fashionable for funding schemes that promote innovative, ground-breaking research. On the other hand, over time it has become clear that mathematics education is a multi-faceted socio-cultural and political endeavor under the influence of many local and global powers. It is therefore not surprising that the field of mathematics education research has expanded so as to include an increasingly wide scope of themes that are at stake, such as the marginalization of particular groups. We therefore highlight Niral Shah’s (USA) response that “historically, these domains of research [content-specific vs socio-political] have been decoupled. The field would get closer to understanding the experiences of minoritized students if we could connect these lines of inquiry.”

Another interesting reflective theme was raised by Nouzha El Yacoubi (Morocco): To what extent can we transpose “research questions from developed to developing countries”? As members of the plenary panel at PME 2019 (e.g., Kazima, 2019; Kim, 2019; Li, 2019) conveyed well, adopting interventions that were successful in one place in another place is far from trivial (cf. Gorard, 2020).

Juan L. Piñeiro (Spain in 2019, Chile in 2020) highlighted that “mathematical concepts and processes have different natures. Therefore, can it be characterized using the same theoretical and methodological tools?” More generally, one may ask if our theories and methodologies—often borrowed from other disciplines—are well suited to the ontology of our own discipline. A discussion started by Niss (2019) on the nature of our discipline, responded to by Bakker (2019) and Cai and Hwang (2019), seems worth continuing.

An important question raised in several comments is how close research should be to existing curricula. One respondent (Benjamin Rott, Germany) noted that research on problem posing often does “not fit into school curricula.” This makes the application of research ideas and findings problematic. However, one could argue that research need not always be tied to existing (local) educational contexts. It can also be inspirational, seeking principles of what is possible (and how) with a longer-term view on how curricula may change in the future. One option is, as Simon Zell (Germany) suggests, to test designs that cover a longer timeframe than typically done. Another way to bridge these two extremes is “collaboration between teachers and researchers in designing and publishing research” (K. Subramaniam, India) as is promoted by facilitating teachers to do PhD research (Bakx et al., 2016).

One of the responding teacher-researchers (Lonneke Boels, the Netherlands) expressed the wish that research would become available “in a more accessible form.” This wish raises the more general questions of whose responsibility it is to do such translation work and how to communicate with non-researchers. Do we need a particular type of communication research within mathematics education to learn how to convey particular key ideas or solid findings? (cf. Bosch et al., 2017)

### Interdisciplinarity and transdisciplinarity

Many respondents mentioned disciplines which mathematics education research can learn from or should collaborate with (cf. Suazo-Flores et al., 2021). Examples are history, mathematics, philosophy, psychology, psychometry, pedagogy, educational science, value education (social, emotional), race theory, urban education, neuroscience/brain research, cognitive science, and computer science didactics. “A big challenge here is how to make diverse experts approach and talk to one another in a productive way.” (David Gómez, Chile)

One of the most frequently mentioned disciplines in relation to our field is history. It is a common complaint in, for instance, the history of medicine that historians accuse medical experts of not knowing historical research and that medical experts accuse historians of not understanding the medical discipline well enough (Beckers & Beckers, 2019). This tension raises the question who does and should do research into the history of mathematics or of mathematics education and to what broader purpose.

Some responses go beyond interdisciplinarity, because resolving the bigger issues such as climate change and a more equitable society require collaboration with non-researchers (transdisciplinarity). A typical example is the involvement of educational practice and policy when improving mathematics education (e.g., Potari et al., 2019).

Let us end this section with a word of hope, from an anonymous respondent: “I still believe (or hope?) that the pandemic, with this making-inequities-explicit, would help mathematics educators to look at persistent and systemic inequalities more consistently in the coming years.” Having learned so much in the past year could indeed provide an opportunity to establish a more equitable “new normal,” rather than a reversion to the old normal, which one reviewer worried about.

## The themes in their coherence: an artistic impression

As described above, we identified eight themes of mathematics education research for the future, which we discussed one by one. The disadvantage of this list-wise discussion is that the entanglement of the themes is backgrounded. To compensate for that drawback, we here render a brief interpretation of the drawing of Fig. 1. While doing so, we invite readers to use their own creative imagination and perhaps use the drawing for other purposes (e.g., ask researchers, students, or teachers: Where would you like to be in this landscape? What mathematical ideas do you spot?). The drawing mainly focuses on the themes that emerged from the first round of responses but also hints at experiences from the time of the pandemic, for instance distance education. In Appendix 1, we specify more of the details in the drawing and we provide a link to an annotated image (available at https://www.fisme.science.uu.nl/toepassingen/28937/).

The boat on the river aims to represent teaching approaches. The hand drawing of the boat hints at the importance of educational design: A particular approach is being worked out. On the boat, a teacher and students work together toward educational and societal goals, further down the river. The graduation bridge is an intermediate educational goal to pass, after which there are many paths leading to other goals such as higher education, citizenship, and work in society. Relations to practices outside mathematics education are also shown. In the left bottom corner, the house and parents working and playing with children represent the link of education with the home situation and leisure activity.

The teacher, represented by the captain in the foreground of the ship, is engaged in professional development, consulting a book, but also learning by doing (cf. Bakkenes et al., 2010, on experimenting, using resources, etc.). Apart from graduation, there are other types of goals for teachers and students alike, such as equity, positive affect, and fluent use of technology. During their journey (and partially at home, shown in the left bottom corner), students learn to orient themselves in the world mathematically (e.g., fractal tree, elliptical lake, a parabolic mountain, and various platonic solids). On their way toward various goals, both teacher and students use particular technology (e.g., compass, binoculars, tablet, laptop). The magnifying glass (representing research) zooms in on a laptop screen that portrays distance education, hinting at the consensus that the pandemic magnifies some issues that education was already facing (e.g., the digital divide).

Equity, diversity, and inclusion are represented with the rainbow, overarching everything. On the boat, students are treated equally and the sailing practice is inclusive in the sense that all perform at their own level—getting the support they need while contributing meaningfully to the shared activity. This is at least what we read into the image. Affect is visible in various ways. First of all, the weather represents moods in general (rainy and dark side on the left; sunny bright side on the right). Second, the individual students (e.g., in the crow’s nest) are interested in, anxious about, and attentive to the things coming up during their journey. They are motivated to engage in all kinds of tasks (handling the sails, playing a game of chance with a die, standing guard in the crow’s nest, etc.). On the bridge, the graduates’ pride and happiness hints at positive affect as an educational goal but also represents the exam part of the assessment. The assessment also happens in terms of checks and feedback on the boat. The two people next to the house (one with a camera, one measuring) can be seen as assessors or researchers observing and evaluating the progress on the ship or the ship’s progress.

More generally, the three types of boats in the drawing represent three different spaces, which Hannah Arendt (1958) would characterize as private (paper-folded boat near the boy and a small toy boat next to the girl with her father at home), public/political (ships at the horizon), and the in-between space of education (the boat with the teacher and students). The students and teacher on the boat illustrate school as a special pedagogic form. Masschelein and Simons (2019) argue that the ancient Greek idea behind school (σχολή, *scholè*, free time) is that students should all be treated as equal and should all get equal opportunities. At school, their descent does not matter. At school, there is time to study, to make mistakes, without having to work for a living. At school, they learn to collaborate with others from diverse backgrounds, in preparation for future life in the public space. One challenge of the lockdown situation as a consequence of the pandemic is how to organize this in-between space in a way that upholds its special pedagogic form.

## Research challenges

Based on the eight themes and considerations about mathematics education research itself, we formulate a set of research challenges that strike us as deserving further discussion (cf. Stephan et al., 2015). We do not intend to suggest these are more important than others or that some other themes are less worthy of investigation, nor do we suggest that they entail a research agenda (cf. English, 2008).

### Aligning new goals, curricula, and teaching approaches

There seems to be relatively little attention within mathematics education research for curricular issues, including topics such as learning goals, curriculum standards, syllabi, learning progressions, textbook analysis, curricular coherence, and alignment with other curricula. Yet we feel that we as mathematics education researchers should care about these topics as they may not necessarily be covered by other disciplines. For example, judging from Deng’s (2018) complaint about the trends in the discipline of curriculum studies, we cannot assume scholars in that field to address issues specific to the mathematics-focused curriculum (e.g., the *Journal of Curriculum Studies* and *Curriculum Inquiry* have published only a limited number of studies on mathematics curricula).

Learning goals form an important element of curricula or standards. It is relatively easy to formulate important goals in general terms (e.g., critical thinking or problem solving). As a specific example, consider mathematical problem posing (Cai & Leikin, 2020), which curriculum standards have specifically pointed out as an important educational goal—developing students’ problem-posing skills. Students should be provided opportunities to formulate their own problems based on situations. However, there are few problem-posing activities in current mathematics textbooks and classroom instruction (Cai & Jiang, 2017). A similar observation can be made about problem solving in Dutch primary textbooks (Kolovou et al., 2009). Hence, there is a need for researchers and educators to align problem posing in curriculum standards, textbooks, classroom instruction, and students’ learning.

The challenge we see for mathematics education researchers is to collaborate with scholars from other disciplines (interdisciplinarity) and with non-researchers (transdisciplinarity) in figuring out how the desired societal and educational goals can be shaped in mathematics education. Our discipline has developed several methodological approaches that may help in formulating learning goals and accompanying teaching approaches (cf. Van den Heuvel-Panhuizen, 2005), including epistemological analyses (Sierpinska, 1990), historical and didactical phenomenology (Bakker & Gravemeijer, 2006; Freudenthal, 1986), and workplace studies (Bessot & Ridgway, 2000; Hoyles et al., 2001). However, how should the outcomes of such research approaches be weighed against each other and combined to formulate learning goals for a balanced, coherent curriculum? What is the role of mathematics education researchers in relation to teachers, policymakers, and other stakeholders (Potari et al., 2019)? In our discipline, we seem to lack a research-informed way of arriving at the formulation of suitable educational goals without overloading the curricula.

### Researching mathematics education across contexts

Though methodologically and theoretically challenging, it is of great importance to study learning and teaching mathematics across contexts. After all, students do not just learn at school; they can also participate in informal settings (Nemirovsky et al., 2017), online forums, or affinity networks (Ito et al., 2018) where they may share for instance mathematical memes (Bini et al., 2020). Moreover, teachers are not the only ones teaching mathematics: Private tutors, friends, parents, siblings, or other relatives can also be involved in helping children with their mathematics. Mathematics learning could also be situated on streets or in museums, homes, and other informal settings. This was already acknowledged before 2020, but the pandemic has scattered learners and teachers away from the typical central school locations and thus shifted the distribution of labor.

In particular, physical and virtual spaces of learning have been reconfigured due to the pandemic. Issues of timing also work differently online, for example, if students can watch online lectures or videos whenever they like (asynchronously). Such reconfigurations of space and time also have an effect on the rhythm of education and hence on people’s energy levels (cf. Lefebvre, 2004). More specifically, the reconfiguration of the situation has affected many students’ levels of motivation and concentration (e.g., Meeter et al., 2020). As Engelbrecht et al. (2020) acknowledged, the pandemic has drastically changed the teaching and learning model as we knew it. It is quite possible that some existing theories about teaching and learning no longer apply in the same way. An interesting question is whether and how existing theoretical frameworks can be adjusted or whether new theoretical orientations need to be developed to better understand and promote productive ways of blended or online teaching, across contexts.

### Focusing teacher professional development

Professional development of teachers and teacher educators stands out from the survey as being in need of serious investment. How can teachers be prepared for the unpredictable, both in terms of beliefs and actions? During the pandemic, teachers have been under enormous pressure to make quick decisions in redesigning their courses, to learn to use new technological tools, to invent creative ways of assessment, and to do what was within their capacity to provide opportunities to their students for learning mathematics—even if technological tools were limited (e.g., if students had little or no computer or internet access at home). The pressure required both emotional adaption and instructional adjustment. Teachers quickly needed to find useful information, which raises questions about the accessibility of research insights. Given the new situation, limited resources, and the uncertain unfolding of education after lockdowns, focusing teacher professional development on necessary and useful topics will need much attention. In particular, there is a need for longitudinal studies to investigate how teachers’ learning actually affects teachers’ classroom instruction and students’ learning.

In the surveys, respondents mainly referred to teachers as K-12 school mathematics teachers, but some also stressed the importance of mathematics teacher educators (MTEs). In addition to conducting research in mathematics education, MTEs are acting in both the role of teacher educators and of mathematics teachers. There has been increased research on MTEs as requiring professional development (Goos & Beswick, 2021). Within the field of mathematics education, there is an emerging need and interest in how mathematics teacher educators themselves learn and develop. In fact, the changing situation also provides an opportunity to scrutinize our habitual ways of thinking and become aware of what Jullien (2018) calls the “un-thought”: What is it that we as educators and researchers have not seen or thought about so much about that the sudden reconfiguration of education forces us to reflect upon?

### Using low-tech resources

Particular strands of research focus on innovative tools and their applications in education, even if they are at the time too expensive (even too labor intensive) to use at large scale. Such future-oriented studies can be very interesting given the rapid advances in technology and attractive to funding bodies focusing on innovation. Digital technology has become ubiquitous, both in schools and in everyday life, and there is already a significant body of work capitalizing on aspects of technology for research and practice in mathematics education.

However, as Cai et al. (2020) indicated, technology advances so quickly that addressing research problems may not depend so much on developing a new technological capability as on helping researchers and practitioners learn about new technologies and imagine effective ways to use them. Moreover, given the millions of students in rural areas who during the pandemic have only had access to low-tech resources such as podcasts, radio, TV, and perhaps WhatsApp through their parents’ phones, we would like to see more research on what learning, teaching, and assessing mathematics through limited tools such as Whatsapp or WeChat look like and how they can be improved. In fact, in China, a series of WeChat-based mini-lessons has been developed and delivered through the WeChat video function during the pandemic. Even when the pandemic is under control, mini-lessons are still developed and circulated through WeChat. We therefore think it is important to study the use and influence of low-tech resources in mathematics education.

### Staying in touch online

With the majority of students learning at home, a major ongoing challenge for everyone has been how to stay in touch with each other and with mathematics. With less social interaction, without joint attention in the same physical space and at the same time, and with the collective only mediated by technology, becoming and staying motivated to learn has been a widely felt challenge. It is generally expected that in the higher levels of education, more blended or distant learning elements will be built into education. Careful research on the affective, embodied, and collective aspects of learning and teaching mathematics is required to overcome eventually the distance and alienation so widely experienced in online education. That is, we not only need to rethink social interactions between students and/or teachers in different settings but must also rethink how to engage and motivate students in online settings.

### Studying and improving equity without perpetuating inequality

Several colleagues have warned, for a long time, that one risk of studying achievement gaps, differences between majority and minority groups, and so forth can also perpetuate inequity. Admittedly, pinpointing injustice and the need to invest in particular less privileged parts of education is necessary to redirect policymakers’ and teachers’ attention and gain funding. However, how can one reorient resources without stigmatizing? For example, Svensson et al. (2014) pointed out that research findings can fuel political debates about groups of people (e.g., parents with a migration background), who then may feel insecure about their own capacities. A challenge that we see is to identify and understand problematic situations without legitimizing problematic stereotyping (Hilt, 2015).

Furthermore, the field of mathematics education research does not have a consistent conceptualization of equity. There also seem to be regional differences: It struck us that equity is the more common term in the responses from the Americas, whereas inclusion and diversity were more often mentioned in the European responses. Future research will need to focus on both the conceptualization of equity and on improving equity and related values such as inclusion.

### Assessing online

A key challenge is how to assess online and to do so more effectively. This challenge is related to both privacy, ethics, and performance issues. It is clear that online assessment may have significant advantages to assess student mathematics learning, such as more flexibility in test-taking and fast scoring. However, many teachers have faced privacy concerns, and we also have the impression that in an online environment it is even more challenging to successfully assess what we value rather than merely assessing what is relatively easy to assess. In particular, we need to systematically investigate any possible effect of administering assessments online as researchers have found a differential effect of online assessment versus paper-and-pencil assessment (Backes & Cowan, 2019). What further deserves careful ethical attention is what happens to learning analytics data that can and are collected when students work online.

### Doing and publishing interdisciplinary research

When analyzing the responses, we were struck by a discrepancy between what respondents care about and what is typically researched and published in our monodisciplinary journals. Most of the challenges mentioned in this section require interdisciplinary or even transdisciplinary approaches (see also Burkhardt, 2019).

An overarching key question is: What role does mathematics education research play in addressing the bigger and more general challenges mentioned by our respondents? The importance of interdisciplinarity also raises a question about the scope of journals that focus on mathematics education research. Do we need to broaden the scope of monodisciplinary journals so that they can publish important research that combines mathematics education research with another disciplinary perspective? As editors, we see a place for interdisciplinary studies as long as there is one strong anchor in mathematics education research. In fact, there are many researchers who do not identify themselves as mathematics education researchers but who are currently doing high-quality work related to mathematics education in fields such as educational psychology and the cognitive and learning sciences. Encouraging the reporting of high-quality mathematics education research from a broader spectrum of researchers would serve to increase the impact of the mathematics education research journals in the wider educational arena. This, in turn, would serve to encourage further collaboration around mathematics education issues from various disciplines. Ultimately, mathematics education research journals could act as a hub for interdisciplinary collaboration to address the pressing questions of how mathematics is learned and taught.

## Concluding remarks

In this paper, based on a survey conducted before and during the pandemic, we have examined how scholars in the field of mathematics education view the future of mathematics education research. On the one hand, there are no major surprises about the areas we need to focus on in the future; the themes are not new. On the other hand, the responses also show that the areas we have highlighted still persist and need further investigation (cf. OECD, 2020). But, there are a few areas, based on both the responses of the scholars and our own discussions and views, that stand out as requiring more attention. For example, we hope that these survey results will serve as propelling conversation about mathematics education research regarding online assessment and pedagogical considerations for virtual teaching.

The survey results are limited in two ways. The set of respondents to the survey is probably not representative of all mathematics education researchers in the world. In that regard, perhaps scholars in each country could use the same survey questions to survey representative samples within each country to understand how the scholars in that country view future research with respect to regional needs. The second limitation is related to the fact that mathematics education is a very culturally dependent field. Cultural differences in the teaching and learning of mathematics are well documented. Given the small numbers of responses from some continents, we did not break down the analysis for regional comparison. Representative samples from each country would help us see how scholars from different countries view research in mathematics education; they will add another layer of insights about mathematics education research to complement the results of the survey presented here. Nevertheless, we sincerely hope that the findings from the surveys will serve as a discussion point for the field of mathematics education to pursue continuous improvement.

## Appendix 1: Explanation of Fig. 1

We have divided Fig. 1 in 12 rectangles called A1 (bottom left) up to C4 (top right) to explain the details (for image annotation go to https://www.fisme.science.uu.nl/toepassingen/28937)

**Table:**

4	- Dark clouds: Negative affect - Parabola mountain	Rainbow: equity, diversity, inclusion Ships in the distance Bell curve volcano	Sun: positive affect, energy source
3	- Pyramids, one with Pascal’s triangle - Elliptic lake with triangle - Shinto temple resembling Pi - Platonic solids - Climbers: ambition, curiosity	- Gherkin (London) - NEMO science museum (Amsterdam) - Cube houses (Rotterdam) - Hundertwasser waste incineration (Vienna) - Los Manantiales restaurant (Mexico City) - The sign post “this way” pointing two ways signifies the challenge for students to find their way in society - Series of prime numbers. 43*47 = 2021, the year in which Lizzy Angerer made this drawing - Students in the crow’s nest: interest, attention, anticipation, technology use - The picnic scene refers to the video *Powers of Ten* (Eames & Eames, 1977)	- Bridge with graduates happy with their diplomas - Vienna University building representing higher education
2	- Fractal tree - Pythagoras’ theorem at the house wall	- Lady with camera and man measuring, recording, and discussing: research and assessment	The drawing hand represents design (inspired by M. C. Escher’s 1948 drawing hands lithograph)
1	Home setting: - Rodin’s thinker sitting on hyperboloid stool, pondering how to save the earth - Boy drawing the fractal tree; mother providing support with tablet showing fractal - Paper-folded boat - Möbius strips as scaffolds for the tree - Football (sphere) - Ripples on the water connecting the home scene with the teaching boat	School setting: - Child’s small toy boat in the river - Larger boat with students and a teacher - Technology: compass, laptop (distance education) - Magnifying glass represents research into online and offline learning - Students in a circle throwing dice (learning about probability) - Teacher with book: professional self-development	Sunflowers hinting at Fibonacci sequence and Fermat’s spiral, and culture/art (e.g., Van Gogh)

## Declarations

In line with the guidelines of the Code of Publication Ethics (COPE), we note that the review process of this article was blinded to the authors.
